# Viral Delivery Systems for CRISPR

**DOI:** 10.3390/v11010028

**Published:** 2019-01-04

**Authors:** Christine L. Xu, Merry Z. C. Ruan, Vinit B. Mahajan, Stephen H. Tsang

**Affiliations:** 1 Edward S. Harkness Eye Institute, New York-Presbyterian Hospital, New York, NY 10032, USA; christinexu787@gmail.com (C.L.X.); zr2187@cumc.columbia.edu (M.Z.C.R.); 2 Jonas Children’s Vision Care and Bernard & Shirlee Brown Glaucoma Laboratory, Columbia University, New York, NY 10032, USA; 3 Omics Laboratory, Byers Eye Institute, Department of Ophthalmology, Stanford University, Palo Alto, CA 94301, USA; mahajanlab@gmail.com; 4 Veterans Affairs Palo Alto Health Care, Palo Alto, CA 94301, USA; 5 Department of Pathology & Cell Biology, Stem Cell Initiative (CSCI), Institute of Human Nutrition, Vagelos College of Physicians and Surgeons, Columbia University, New York, NY 10032, USA

**Keywords:** CRISPR, viral vectors, gene therapy, animal models, gene editing

## Abstract

The frontiers of precision medicine have been revolutionized by the development of Clustered Regularly-Interspaced Short Palindromic Repeats (CRISPR)/Cas9 as an editing tool. CRISPR/Cas9 has been used to develop animal models, understand disease mechanisms, and validate treatment targets. In addition, it is regarded as an effective tool for genome surgery when combined with viral delivery vectors. In this article, we will explore the various viral mechanisms for delivering CRISPR/Cas9 into tissues and cells, as well as the benefits and drawbacks of each method. We will also review the history and recent development of CRISPR and viral vectors and discuss their applications as a powerful tool in furthering our exploration of disease mechanisms and therapies.

## 1. Introduction

The development of Clustered Regularly-Interspaced Short Palindromic Repeats (CRISPR)/Cas9 into a genetic editing tool has revolutionized the field of precision medicine [1]. To date, CRISPR/Cas9 has been used to develop animal models, understand disease mechanisms, and validate treatment targets [2,3,4,5]. The greatest potential application of CRISPR/Cas9 is gene therapy via the correction of disease-causing mutations. Before CRISPR emerged as a tool for efficient and specific gene editing, gene therapies were largely limited to gene augmentation therapies, which correct disease symptoms and signs secondary to loss-of-function alleles by adding functional alleles [6,7]. However, it is difficult to control the dose of the added alleles. More importantly, many disorders are caused by genes with sizes that exceed the packaging capacities of viral vectors, making it impossible to add full-length alleles as a method to correct the underlying disorder [8,9]. Researchers have explored multiple viral vectors and delivery methods in order to expand the carrying capacities and the spectrum of disorders that can be treated. Modification of adenoviral vectors has expanded the carrying capacity to as high as 35 kb [10,11]. However, diseases caused by gain-of-function mutations or dominant negative alleles cannot be corrected by gene augmentation therapies. Genetic editing with CRISPR/Cas9 expands the application of genome surgery because it offers the possibility of treating diseases caused by both loss-of-function mutations and gain-of-function mutations, regardless of gene size. In this review, we will introduce the basics of CRISPR/Cas9 and commonly used viral delivery systems, summarize the recent work performed with the most cutting-edge viral technology, and discuss the future directions of these tools.

## 2. Viral Delivery Systems

Many viral delivery systems have been used for delivery of CRISPR-Cas9. A brief summary of the viral vectors is shown in Table 1.

### 2.1. Adeno-Associated Viruses (AAV)

Although there are many classes of viral vectors, adeno-associated viruses (AAVs) have largely been used for CRISPR genome editing. The reasons why AAVs are the most popular vectors are multifold. First, AAVs have already been approved for a number of human clinical trials in gene augmentation therapies due to their good safety profile and therapeutic potential [12]. AAVs are significantly less immunogenic than other viruses [13]. Furthermore, they elicit only a mild toxicity in animal models at high doses [12]. After transduction, AAV DNA remains mostly episomal besides integrating into hotspots in mitochondrial DNA and a specific location on chromosome 19 termed AAVS1 [14,15,16]. Both integration sites are currently considered safe and do not mediate tumorigenesis. Some studies have taken advantage of the integrations to enable long term gene expression in dividing cells [17]. Another advantage of AAVs is that AAV concameters can deliver stable transgene expression because they exist long-term in non-dividing cells. A large number of AAV serotypes also allow for tissue-targeted gene delivery, as various serotypes have been shown to be suitable for CRISPR-editing in specific tissues (e.g., AAV9 for murine brain and AAV6 for murine muscle).

#### 2.1.1. ssAAV vs. dsAAV

AAV vectors contain single-stranded DNA (ssDNA), which must be made transcriptionally active via the conversion into a double-stranded template (dsDNA) [18]. This process can be carried out through two mechanisms: (1) two viruses co-infecting the same cell and the subsequent annealing of plus and minus strands or (2) synthesis of the second strand of DNA de novo in the infected cell. Since both processes are rate-limiting steps in transgene expression, Wang et al. tested AAVs, which solely package hairpin-like dsDNA, and found that they could lead to improved gene transfer both in vitro and in vivo. A significant drawback, however, of the dsAAV is that less foreign DNA can be packaged into dsAAV than ssAAV.

#### 2.1.2. Dual AAV

Due to their relatively low immunogenicity and high efficiency compared to other viral vectors, AAVs have been popular vectors for gene therapy research [19]. The carrying capacity of 4.7 kb, however, limits single AAV capsids from packaging large genes. In order to overcome this obstacle, researchers have modified their AAV delivery strategy and created dual AAV systems, in order to expand the capacity to approximately 9 kb [20]. There are four methods of dual AAV systems: fragmented, overlapping, trans-splicing, and hybrid.

The fragmented AAV dual vector system requires truncating transgenes at undefined points and then packaging these incomplete fragments into separate AAVs. When strands of DNA with overlapping regions of the transgene undergo homologous recombination (HR), the resulting product is the complete target transgene. Although this method has led to successful transgene expression in numerous studies, such as one by Lopes et al. which utilized dual AAV to deliver *MYO7A* cDNA into the eyes of *Myo7a*-null mice, this approach still has its disadvantages [21]. For example, researchers cannot control transgene packaging, because truncation of transgenes occurs at undefined points. Furthermore, due to the system’s use of a mixed population of AAV vectors containing various truncated fragments of the plus and minus transgene strands, there is a strong likelihood that numerous unwanted transgene products are produced in addition to the target transgene.

The overlapping AAV dual vector system contains two transgenes with a designated overlap in order to induce HR post-transfection. They are flanked on both ends by an inverted terminal repeat sequence (ITR), a sequence found in AAVs which mediate AAV genome recombination [20]. After the dual AAVs co-infect the same cell, the ITRs from the transgenes induce tail-to-head concatemerization of the transgene. Following concatemerization, HR will occur between the overlapping regions. Unlike the fragmented approach, overlapping AAV transgenes carry a defined portion of the therapeutic gene. Halbert et al. demonstrated that this strategy could successfully split an alkaline phosphatase (AP) gene into AAV2 and AAV6 capsid proteins. Mice airways were delivered dual vectors and the AAVs were able to transduce lung cells almost as well as an intact vector with single gene delivery.

In the trans-splicing AAV dual vectors, there is no region of overlap. Instead, the fragments of transgene are separated by ITRs. The ITRs mediate the joining of 5′ and 3′ genomes via tail-to-head concatemerization [20,22]. Endogenous splicing mechanisms mediate the removal of the ITR and the successful joining of transgene fragments [19]. A risk of this approach, however, is that the transgene fragments could join incorrectly.

Hybrid AAV dual vectors, first discussed in 2008 by Ghosh et al., contain an overlapping region and splice donor/splice acceptor sites, thereby functioning as a combination of the overlapping and trans-splicing approaches [23]. The intein-split provides double the opportunity for transgene regeneration. The final desired transgene product is created after vector co-infection, concatemerization, and a combination of splicing (induced by the splice donor/acceptor sites) and HR (induced by the overlapping regions) [20]. In the study by Ghosh et al., the hybrid method performed better than both the overlapping and trans-splicing methods.

#### 2.1.3. Dual AAV and CRISPR

Currently, researchers most frequently employ a dual AAV system for CRISPR-mediated gene correction: one AAV expresses Cas9 while the other expresses the sgRNA and donor template DNA [24]. The commonly used SpCas9 is ~4.2 kb, so a single AAV (~4.5 kb) is not sufficient to fit all the necessary CRISPR components [25]. For example, in 2016, Yang et al. used the dual AAV approach with *Staphylococcus aureus* Cas9 (SaCas9) to deliver CRISPR constructs into newborn mice with partially deficient ornithine transcarbamylase [24], a urea cycle enzyme in the liver. After AAV delivery, these mice experienced a reversion of the mutation in 10% of their hepatocytes. Furthermore, there was an increase in survival of mice with a high-protein diet, a stressor which exacerbates this in-born error of metabolism. This is not only significant because it suggests that in vivo genome editing was efficacious in reversing a lethal disease in an animal model for metabolic disease, but also, because delivering dual AAVs in newborns reduces the potential for immunological adverse events. This delivery method causes SaCas9 protein expression to be transient, because non-integrated vectors are diluted when hepatocytes proliferate.

In 2015, Swiech et al. used a dual-vector system to target *Mecp2* in vivo in the adult mouse brain [26]. MeCP2 is a methyl CpG binding protein that contributes to Rett syndrome when mutated. A deficiency in MeCP2 is linked to neuronal morphological and electrophysiological defects. After delivering separate AAVs that carried SpCas9 (AAV-SpCas9) and sgRNA (AAV-SpGuide), they confirmed the successful knockdown of *Mecp2* via immunostaining, quantification of MeCP2 positive cells using DAPI staining in the dentate gyrus (DG), and Western blot analysis of MeCP2 two weeks post-injection. Given that MeCP2 plays a crucial role in learning, they used a behavioral test, the contextual fear-conditioning (CFC) paradigm test, to determine that the CRISPR-treated *Mecp2* knockdown mice exhibited impairments in their contextual memory.

Swiech et al. also used dual AAV vectors for multiplex gene editing. They built an AAV-SpGuide carrying three separate sgRNAs to target *Dnmt1*, *Dnmt3a*, and *Dnmt3b*, and they delivered this AAV-SpGuide along with AAV-SpCas9 into the DG of adult mice. All three loci harbored indels, with a rate of 75% in *Dnmt1* and *Dnmt3a* and 50% in *Dnmt3b*. Using targeted sequencing, they discovered that the biallelic modification rate for transduced cells was over 60% for *Dnmt1*, 42% for *Dnmt3a*, and 17% for *Dnmt3b*. Out of all transduced neurons, roughly 62% had indels in both *Dnmt1* and *Dnmt3a* and 35% had simultaneous indels in all three genes. The triple DNMT knockdown mice exhibited impaired memory formation in the CFC test “trained context” condition, but no phenotype was found for the open field, elevated plus maze, and novel object recognition tests. This suggests that the multiplex genome editing was able to produce knockdown in only a fraction of targeted cells.

In 2017, Bak et al. demonstrated that a dual AAV approach could be used to integrate large transgenes into endogenous DNA using CRISPR [27]. Since creating double-stranded breaks with Cas9 induces HR when there is a homologous donor DNA template, they harnessed this method to mediate targeted transgene integration. AAV vectors with the first part of the transgene (“Donor A”) contained the sgRNA target site, so that it would be maintained even after HR. The researchers called the AAV vector with the second part of the transgene “Donor B.” Cas9 mRNA was co-electroporated into cells, and once expressed, it induced double-stranded cuts to the target site. Two sequential rounds of HR allowed the full transgene to be integrated at the site: Donor A was incorporated first due to homology arms for the target locus, and Donor B was incorporated due to “stuffer DNA,” a homology arm for Donor B. This method was successful in the K562 cell line, primary human T cells, and CD34+ hematopoietic stem and progenitor cells.

#### 2.1.4. Triple AAV

Although the dual AAV approach overcomes the limitations of a single AAV’s small carrying capacity, some genes still exceed the maximum 9 kb allowed through the dual AAV method. For example, the *CDH23* gene (involved in Usher 1D) measures 10.1 kb and the *DMD* gene (involved in muscular dystrophy) measures 11.1 kb [20]. For packaging genes that exceed the carrying capacity of dual vectors, researchers have developed a triple AAV approach.

In 2014, Koo et al. used the trans-splicing approach with triple AAVs to deliver the human *DMD* coding sequence to dystrophic *mdx* mice [28]. Three separate AAVs carried sequential exons of *DMD* and the full-length gene was joined together upon ITR-mediated trans-splicing. Cross-sectioning and staining of the sarcolemma confirmed that the full-length human dystrophin protein was expressed in the mouse’s muscle fibers.

In 2018, Maddalena et al. tested retinal gene transfer using triple AAVs to study Usher syndrome type 1D caused by mutations in *CDH23* and Alström syndrome type I caused by mutations in *ALMS1* [20]. Transcriptomic analysis demonstrated that triple *CDH-* and *ALMS-*AAV vectors could efficiently transduce HEK293 cells. Mice photoreceptors transduced by triple AAV vectors were shown to have a transduction efficiency of 4% and the proper localization of ALMS1 2 months after injection. In the pig retina, a good model because it is similar in size to the human retina, transduction levels of triple AAV vectors could reach 40% of those seen with single AAV vectors.

### 2.2. Adenoviral Vectors (AdV)

The adenovirus (AdV) is a non-enveloped, double-strand DNA virus with an icosahedral nucleocapsid. It is capable of infecting both dividing and non-dividing cells. Its genome, which usually ranges from 34–43 kb long, is flanked by two ITR sequences. The AdV genome remains extrachromosomal following injection and does not integrate into the host genome [29]. As a delivery vector for CRISPR/Cas9-based gene editing, this limits potential off-target effects, which are unwanted mutations at sites other than the designated target site.

Due to high transduction efficiency and minimal human symptoms, numerous efforts have been made to optimize the AdV as a gene delivery vector. First generation recombinant AdV vectors are devoid of viral gene E1 [30]. These vectors cause an acute as well as chronic immune response. The former is caused by the viral capsid, while the latter is triggered by viral gene expression. Thus, second generation vectors have been developed to attenuate chronic immune responses through the deletion of viral gene E2 and E4. The packaging capacities of these vectors are around 8 kb [31,32]. The latest generation of AdV vectors, helper-dependent, or gutless AdV vectors, are devoid of all viral genes. These vectors only contain ITRs and encapsidation (ψ). This allows up to 35 kb packaging capacity, which makes it ideal for delivery of the entire CRISPR/Cas9 system in one vector [10,11]. In addition, these vectors do not elicit chronic immune responses.

However, acute phase immune responses might still be elicited by the viral capsid. In addition, most humans have been infected with AdV since infancy and have generated neutralizing antibodies at least towards common serotypes of AdV [29,33]. Maximum serum concentration of genes in gene augmentation therapy gradually decline to about 10% in 24 months due to acute phase immune responses and neutralizing antibodies, thus hampering the efficacy of gene augmentation therapy [34].

AdV delivery of CRISPR/Cas9 has been used in establishing disease models, developing tools for drug discovery, and treating existing diseases. To establish disease models, in 2014, Maddalo et al. generated a model of non-small cell lung cancer by creating an *Eml4* and *Alk* fusion gene through the intratracheal instillation of AdV-delivered CRISPR/Cas9 [35]. In 2015, Wang et al. developed a mouse model mimicking Nonalcoholic steatohepatitis (NASH) using AdV-delivered SpCas9 targeting *Pten*. The NASH phenotype resulted from the *Pten* mutation as well as humoral and cellular immunity against SpCas9 [36].

To demonstrate the potential of CRISPR/Cas9 for drug discovery, Voets et al. used AdV to inactivate the *SMAD3* gene in normal human lung fibroblasts and bronchial epithelial cells with multiplicity of infection as low as 20 [37].

In the realm of treating existing diseases, Ding et al. reported inducing loss-of-function mutation in *PCSK9* in mouse livers to decrease their plasma cholesterol levels [38]. Efforts have been successful in restoring muscle function in vitro in Duchenne muscular dystrophy patient-derived muscle progenitor cells [39] and in vivo in mdx mice [40,41]. Li et al. created HIV-resistant primary CD4+ T cells by adding cell membrane *CCR5*Δ32 variant using AdV-delivered CRISPR/Cas9 [42].

### 2.3. Lentiviral Vectors (LV)

The lentivirus (LV) is a single-stranded (ss) RNA spherical virus. Similar to the AdV, it transduces both dividing and non-dividing cells. The latest LV systems split essential genes into 3 plasmids, thus reducing the likelihood of producing viable viral particles within cells. The packaging capacity of the LV is around 8 kb [43].

One great advantage of the LV is its ability to be pseudotyped with other viral proteins. This allows for the engineering and altering of the LV’s cellular tropism [44]. In addition, LV vectors are deleted of all the viral genes and do not activate the immune system [45,46].

As a retrovirus, however, it integrates into the host genome, which can be an advantage as a delivery vector in gene augmentation therapies involving dividing cells. For CRISPR/Cas9 delivery, however, host genome integrations could lead to unwanted off-target insertional mutagenesis and do not benefit genome surgery [46,47]. A non-integrating LV was engineered to avoid unwanted integration. In these vectors, selected mutations were induced within the integrase coding region to eliminate the integrase activities without affecting reverse transcription and transport of the pre-integrating complex into the nucleus [48,49,50].

Since most labs do not have the capacity to generate the non-integrating LVs, LV vectors are used less often than AAVs and AdVs, and LVs are most commonly used to create disease models. In 2014, multiple labs developed genome-wide loss-of-function genetic screening approaches suitable for both positive and negative selection using a sgRNA library in human and mouse cells [51,52,53].

Around the same time, Koike-Yusa et al. developed a similar screening approach in mouse embryonic stem cells [54]. These studies have already enabled identification of new therapeutic targets and disease mechanisms including the identification of the genes essential for the West-Nile-Virus induced cell death pathway [55,56]. In addition to genetic screening, mouse models were generated with LV-delivered CRISPR-Cas9. A great example is the mouse model of acute myeloid leukemia (AML) generated by modifying up to five genes in a single hematopoietic stem cell to mimic the genetic complexity of malignancy [57].

### 2.4. Bacteriophages

Bacteriophages are a group of viruses that infect bacteria or archaea. They consist of many families of viruses that vary in structure and target organisms. The idea of using bacteriophages in combating multi-drug-resistant bacteria has been appreciated by the scientific community for many years. However, the development of phage therapy was limited by the specific susceptivity of bacteria [58]. This has changed drastically after the combination of bacteriophage and CRISPR-Cas9. In 2014, two groups pioneered using bacteriophages to deliver RNA-guided nucleases targeting specific virulence and resistance genes to modulate complex bacterial populations. Citorik et al. reported improved survival of a *Galleria mellonella* infected model while Bikard et al. specifically killed virulent *Staphylococcus aureus*, while sparing and immunizing the non-virulent *Staphylococcus aureus* in the mouse model [59,60]. Later, Yosef et al. further developed this technology with lytic phage to selectively kill multi-drug-resistance bacteria [61]. In 2017, the same group developed a generalizable platform to create customizable bacteriophages which enhance DNA transfer to hosts that were previously difficult to infect [62].

### 2.5. Non-Viral Delivery

Non-viral delivery methods have also been used to deliver CRISPR/Cas9. These include naked DNA, DNA encapsulating liposomes, compacted DNA nanoparticles (e.g., gold, lipid), and cell-penetrating peptides. Generally, these methods carry low risk of immune responses commonly associated with viral delivery [44,63,64]. However, due to difficulties in yielding proper ratio for gene editing and the lack of transduction efficacy, these methods lack the potential to deliver CRISPR/Cas9 in clinical settings in the near future [65].

## 3. CRISPR

Researchers currently employ a wide variety of Cas9 proteins from different bacterial strains in order to best suit their experimental needs. Table 2 summarizes the strains of Cas9 discussed below.

### 3.1. SpCas9

Cas9 from *Streptococcus pyogenes* (SpCas9) is a popular choice for CRISPR experiments [5]. SpCas9 can recognize a 5′-NGG protospacer adjacent motif (PAM), which is part of the non-self-sequence recognized by the CRISPR bacterial adaptive immune system. PAM is the essential targeting component that binds Cas9 [66,67,68]. This translates to a targetable site approximately every 8 bp in the human genome. Furthermore, SpCas9 has been known to successfully carry out multiplex genome editing (CRISPR editing at multiple target sites) in mammalian cells through the aid of multiple single guide RNAs (sgRNAs) [67]. When using the SpCas9 for experiments, researchers typically use the dual AAV system to administer the CRISPR constructs, since the SpCas9 gene itself measures 4.2 kb (while the AAV has a carrying capacity of 4.5 kb) [25].

### 3.2. SaCas9

Observing the limitations of SpCas9 due to its large size, researchers have been turning to Cas9 from other bacterial strains in order to accommodate the limitations imposed by the single AAV delivery approach. The Cas9 from *Staphylococcus aureus* (SaCas9) is over 1 kb shorter (3.15 kb) than SpCas9 [69]. SaCas9 recognizes PAMs that are 5′-NNGRRT (R = purine). Ran et al. packaged SaCas9 and sgRNA designed to target proprotein convertase subtilisin/kexin type 9 (*Pcsk9*) into single AAV vectors. *Pcsk9* binds to the receptor of low-density lipoprotein (LDL) particles which carry cholesterol. Inhibitors of Pcsk9 effectively lower circulating LDL, thus protecting against vasculopathies [70]. Following tail vein injection, mouse whole liver tissues exhibited insertion-deletion (indel) formation at rates greater than 40%. Furthermore, levels of Pcsk9 decreased in the serum by 95% and cholesterol levels decreased by 40%, levels which were sustained even after four weeks post-injection. Off-targeting analysis with direct in situ breaks labeling, enrichment on streptavidin and next generation sequencing (BLESS) demonstrated that SaCas9 performs genome editing with high specificity in vivo [71].

### 3.3. CjCas9

In February 2017, Kim et al. presented efficient in vivo genome editing using the smallest Cas9 orthologue (2.95 kb) that has been characterized as of February 2017: *Campylobacter jejuni* (CjCas9) [72]. Fonfara et al. previously reported the optimal in vitro PAM site for CjCas9 to be 5′-NNNNACA, and by testing a variety of PAM sites, Kim et al. determined that CjCas9 could generally cleave target sites with the PAM site 5′-NNNNRYAC (where Y = pyrimidine and R = purine) [72,73]. Specifically, they found that the optimal PAM site was 5′-NNNNACAC. Using the off-targeting analysis platform Digenome-Seq allowed them to discover that CjCas9 had high specificity without compromising lower editing efficiency.

Next, they utilized single AAVs to perform in vivo genome editing with CjCas9. Muscle-tropic AAV serotype 9 vectors (AAV9s) carrying muscle-specific *Spc512* promoter-driven CjCas9 and sgRNA targeting *Rosa26* were injected intramuscularly into the tibialis anterior muscles of C57BL6J mice. Indels induced by CjCas9 were detected at 8 weeks and 32 weeks after injection with a frequency of approximately 17% and 13%, respectively. Furthermore, long-term expression of CjCas9 was observed without any detectable off-target effects at 20 potential sites.

Furthermore, they tested the therapeutic potential of CjCas9 by targeting the *Vegfa* or *Hif1a* genes, genes associated with choroidal neovascularization (CNV) and age-related macular degeneration (AMD) in mice. After intravitreal injection, they observed that *Vegfa-* or *Hif1a-*specific CjCas9 (AAV-CjCas9: *Vegfa* or AAV-CjCas9: *Hif1a*) induced indels at a frequency of approximately 20% and 58%, respectively. They also discovered decreased VEGFA protein levels in the retina after treatment with *Vegfa-* or *Hif1a-*specific CjCas9, but not *Rosa26*-specific CjCas9. In RPE cells treated with AAV-CjCas9: *Vegfa*, there was a decrease in VEGFA protein level. Since this was not observed with AAV-CjCas9: *Hif1a*, it suggests a differential regulation of VEGFA expression in the retina and RPE cells. Finally, they tested the potential for CjCas9 to treat choroidal neovascularization (CNV). Six weeks after injecting either AAV-CjCas9: *Vegfa* or AAV-CjCas9: *Hif1a*, they used laser treatment to induce CNV. One week later, they found that both AAV-CjCas9: *Vegfa* and AAV-CjCas9: *Hif1a* reduced the CNV area by approximately 24% and 20%, respectively. These experiments suggest that CjCas9 can carry out in vivo genome editing and that it has the potential to treat AMD.

### 3.4. Cas12a

Cas12a (type-V Cpf1) from *Lachnospiraceae bacterium ND2006* (LbCpf1) and *Acidaminococcus sp. BV3L6* (AsCpf1) have many differences from SpCas9, but they are gaining popularity because they have higher targeting specificity in mammalian cells, as measured through Digenome-Seq analysis [74,75]. Cas12a enzymes are single crRNA-guided endonucleases measuring 3.9 kb, and their distinguishable differences from Cas9 include: a T nucleotide-rich PAM site (5′-TTTV), self-catalysis of crRNA maturation, and a staggered dsDNA cut [76,77,78,79]. The staggered cut produces “sticky ends”, which are conducive for inducing gene insertion via non-homologous end joining (NHEJ) [76]. Programming the sticky end sequence allowed researchers to perform site-specific NHEJ-directed genome editing that would overcome challenges incurred by homology-directed repair (HDR), which is only feasible in dividing cells. For example, Zetsche et al. found that AsCpf1 and LbCpf1 were able to robustly target and edit the *DNMT1* gene in human HEK293FT cells.

## 4. Clinical Use and Conclusions

To date, clinical trials that involve CRISPR/Cas9 target malignancies, Mendelian hematological disorders, and HIV with cells modified by CRISPR/Cas9 in vitro. None of the trials currently registered on clinicaltrials.gov use viral delivery systems in combination with CRISPR/Cas9 [80]. However, there are currently 44 completed and 98 active clinical trials with AAV, mostly gene augmentation therapy to treat diseases caused by loss-of-function mutations. There are more than 140 active trials in which AdVs were used as oncolytic virus, vaccines, or delivery vectors for functional alleles. More than 500 active trials involve lentivirus mainly used as gene delivery tools for ex vivo gene therapy with hematopoietic stem cells. The major barrier of clinical use of CRISPR/Cas9 is its off-target effects [81,82,83]. Clinical trials involving CRISPR genome editing should only be performed with the increase in specificity of CRISPR/Cas9 and the proven safety profile of viral vectors.

## Figures and Tables

**Table 1:** Summary of different delivery vectors.

	Packaging Capacity	Genetic Material	Vector Genome Form	Key Features
AAV	4.7 kb	ssDNA	Mainly episomal	Gene augmentation therapy of small genes in human clinical trials. Gene editing in cells and animal models. Oftentimes, more than one vector is needed due to limited packaging capacity.
AdV	35 kb	dsDNA	Episomal	Cancer treatment in human clinical trials. Proof-of-principle in gene editing in cells and animal models
LV	8 kb	ssRNA	Integrated	Infect dividing cells without transgene dilution. Unwanted off-target effect when combined with CRISPR-Cas9. Mainly used to develop screening tools.
Bacteria Phage	Varies	Varies	N/A	Mainly used in anti multi-drug-resistance bacteria

N/A = Not applicable.

**Table 2:** Summary of CRISPR/Cas9 systems.

Cas9 Strain	PAM	Size	Type of Cut	Citation
SpCas9	5′-NGG	4.2 kb	Blunt	[5,25,66,67,68]
SaCas9	5′-NNGRRT	3.15 kb	Blunt	[69,70,71]
CjCas9	5′-NNNNRYAC	2.95 kb	Blunt	[72,73]
Cas12a	5′-TTTV	3.9 kb	Staggered	[74,75,76,77,78,79]

R = purine, Y = pyrimidine. V = Not U/T (A or C or G).
